# A Comprehensive Analysis of Skin Cancer Concerns and Protective Practices in Manitoba, Canada, Highlights Lack of Skin Cancer Awareness and Predominance of High-Risk Sun Exposure Behaviors

**DOI:** 10.3390/cancers16173093

**Published:** 2024-09-05

**Authors:** François Lagacé, Santina Conte, Lorena A. Mija, Amina Moustaqim-Barrette, Farhan Mahmood, Jonathan LeBeau, Alyson McKenna, Mahan Maazi, Johnny Hanna, Alexandra Sarah Victoria Kelly, Elham Rahme, Travis J. Hrubeniuk, Sandra Peláez, Ivan V. Litvinov

**Affiliations:** 1 Division of Dermatology, McGill University, Montreal, QC H4A 3J1, Canada; 2 Faculté de médecine, Université de Montréal, Montréal, QC H3T 1J4, Canada; 3 Faculty of Medicine, McGill University, Montreal, QC H4A3J1, Canada; 4 Faculty of Medicine, University of British Columbia, Vancouver, BC V6T 1Z4, Canada; 5 Faculté de médecine, Université Laval, Québec, QC G1V 0A6, Canada; 6 Faculty of Sciences, University of Ottawa, Ottawa, ON K1N 6N5, Canada; 7 Division of Clinical Epidemiology, McGill University, Montreal, QC H4A 3J1, Canada; 8 Population Oncology, Cancer Care Manitoba, Winnipeg, MB R3C 2B1, Canada; 9 Community Health Sciences, Max Rady College of Medicine, University of Manitoba, Winnipeg, MB R3C 2B1, Canada; 10 School of Kinesiology and Physical Activity Sciences, Faculty of Medicine, University of Montreal, Montreal, QC H3T 1J4, Canada; 11 Research Centre, Sainte-Justine University Hospital, Montreal, QC H3T 1C5, Canada

**Keywords:** melanoma, skin cancer, skin cancer prevention, sun protection, skin cancer awareness, risk factors, UV exposure, Canadian health, prairies

## Abstract

**Simple Summary:**

Skin cancer rates in Canada are rising quickly, with about one-third of Canadians likely to be affected in their lifetime. Despite this alarming trend, government actions to reduce skin cancer are limited. Our study, conducted in Manitoba, found that many residents have risky sun exposure habits and lack awareness about skin cancer. Over 65% reported a history of sunburns, more than half had used tanning beds, and a large majority recently tanned for pleasure. Misconceptions are common, with over 50% believing that tans are healthy or a sign of beauty. Moreover, sun protection practices are inadequate, with less than 60% using protective clothing and under 50% using sunscreen. These findings highlight the need for targeted public health campaigns to improve awareness and promote better sun protection behaviors to prevent future skin cancers in Manitoba.

**Abstract:**

The rapidly increasing skin cancer rates in Canada are alarming, with current data estimating that 1/3 of Canadians will be affected in their lifetime. Thus, deeper understanding of high-risk sun exposure behaviors is needed to help counter this trend. Only limited action has been taken by federal/provincial governments to reduce skin cancer incidence. A cross-sectional survey study was conducted in Manitoba, with frequency counts, means, and percentages used to encapsulate responses. Age- and gender-adjusted odds ratios were calculated using logistic regression analyses. Our study identified worrying inadequacies in sun protective behaviors and attitudes, with the threat of such high-risk behaviors amplified by a lack of skin cancer awareness. Alarming elements were noted in participants’ sun exposure history (>65% reported a history of sunburns, >50% previously used a tanning bed, and >75% recently tanned for pleasure), beliefs and attitudes (>50% believe that they look better/healthier with a tan, and >40% believe that having a base tan is protective against further sun damage), and sun protection efforts (sun protective clothing was used <60% of the time, sunscreen was used by <50%, and there was a lack of knowledge about sunscreen characteristics in ~30% of respondents), in addition to significant differences being established between demographic subgroups (based on gender, age, skin phototype, income, and education attained). This study provides worrisome insight onto the grim landscape of sun protective behaviors and attitudes in Manitoba, which will inevitably translate into higher skin cancer rates and should serve as a call to action to promote targeted public health messaging in this jurisdiction and beyond.

## 1. Introduction

In recent decades, national Canadian trends have noted an increase in cutaneous melanoma (CM) incidence rates, while CM-associated mortality rates have been on the decline since 2013 [1,2,3,4,5,6]. The decline in mortality rates coincides with the introduction of immunotherapy and targeted therapies for the treatment of CM, which have been approved for use in Canada since 2012 [7]. A similar trend was observed in the United States, where treatment advances have led to decreasing mortality rates since 2013 [8,9]. Advances in diagnostic techniques, such as dermoscopy, have led to earlier detection of CM, which has also contributed to the notable decline in mortality rates [10]. Despite this, ultraviolet (UV) exposure remains a significant factor driving these concerning incidence trends. Unfortunately, there have been only limited concerted efforts on the part of Canadian federal and provincial governments to improve the landscape of skin cancer across the country through public health initiatives, legislation, or guidelines [11]. Fortunately, CM morbidity and mortality can be prevented through patient education, sun protection, regular screening, and public health campaigns, as exemplified by Australia’s successful efforts to reduce skin cancer rates across their country in the last several decades [12,13,14,15].

In the Canadian context, CM’s incidence rate per 100,000 individuals increased from 12.29 between 1992 and 2010 to 20.75 between 2011 and 2017 [1]. Provincial differences with regard to incidence were identified, with Prince Edward Island and Nova Scotia having notably higher age-standardized incidence rates compared with the national average (30.94 and 27.76, respectively), while Manitoba (16.99) and Saskatchewan (15.14) each had lower rates between 2011 and 2017. However, despite lower CM incidence in Manitoba, when evaluating all provinces/territories, Manitoba was found to have the highest mortality to incidence ratio (MIR) in Canada (Manitoba MIR, 19.9%; Canadian rate, 15.4%), which could signify deficiencies in healthcare delivery or a lack of patient awareness or determination to receive care in the province [16].

The factors underlying variations in skin cancer incidence and mortality across Canada remain to be fully elucidated. While studies by the Western Canada Melanoma Study Group have begun to elucidate important relationships between demographic factors and CM incidence, these studies are now significantly dated. For instance, these studies revealed a positive gradient of CM risk amongst socioeconomic classes, with a significant inverse association between farming and CM risk [17], a positive correlation between CM risk and levels of physical activity [18], and the possibility of chronic or long-term occupational sun exposure, surprisingly, being protective against CM [19]. In the early 2010s, the Manitoba Youth Health survey collected data from students in grades 7 through 12, reporting that the act of indoor tanning was associated with age, part-time work, physical activity, and many consumption behaviors and lifestyle risk factors [20]. Importantly, studies conducted across the globe have noted that sunbed use expanded rapidly in the 1980s and likely contributed to the increase in CM incidence that we can observe today [21,22,23]. 

With the notable increase in CM cases over recent decades, this study aims to examine UV exposure, sun protective behaviors, levels of concern regarding CM, and baseline CM knowledge in Manitoba, Canada. This region has received limited attention in existing literature, and it was preferentially selected owing to our partnership with the Manitoba Tomorrow Project, which facilitated data collection. Furthermore, we aimed to identify variations among various demographic groups, assessing by gender, skin phototype, education, income, and age. Our overarching objective was to gain a comprehensive understanding of sun protective behaviors and knowledge in Manitoba, to thereby facilitate the development of targeted public health campaigns and initiatives.

## 2. Materials and Methods

The study design adheres to the guidelines outlined in the Checklist for Reporting Results of Internet E-Surveys (CHERRIES) [24].

### 2.1. Study Design

A cross-sectional study was carried out in Manitoba through survey administration. A sample size of 385 individuals was deemed adequate based on calculations using a 95% confidence interval and a 5% margin of error. To allow for stratification of variables by different parameters, we set a target of at least 385 participants per stratum. In total, 3347 participants were included in the final analyses.

### 2.2. Ethics Statement

The study protocol received approval from both the Research Ethics Board of McGill University (study number A04-B16–20B) and the Manitoba Tomorrow Project’s review committee. All participants provided informed consent electronically before completing the survey.

### 2.3. Development and Pre-Testing 

An electronic validated patient questionnaire, the Sun Exposure and Behavior Inventory (SEBI) [25], was sent to participants. Additional questions were added to the questionnaire to render it more complete for our study purposes, including more demographic variables and detailed questions on UV exposure, sun protection practices, CM knowledge, and level of worry about CM. The clarity, objectivity, and directness of the survey questions were ensured through collaboration with MaelStrom Research, a multidisciplinary team that aids in implementing comprehensive data frameworks for epidemiological studies [26]. Finally, the survey underwent stringent testing by multiple trial users prior to participant recruitment to ensure that there were no technical issues. 

### 2.4. Recruitment Process and Survey Administration 

Voluntary recruitment of participants took place between August 2021 and March 2024, which was performed in conjunction with the Manitoba Tomorrow Project as of August 2023. The Manitoba Tomorrow Project is part of and funded by the Canadian Partnership for Tomorrow’s Health (CanPath) initiative, which aims to better understand the causes of cancer and other chronic diseases, thus aligning with our study goals [27]. Participants were recruited through the following modalities: advertisement, workspace and community events, community leaders promoting participation, outreach activities, and media coverage. 

For this study, we initially sent email invitations to members of the Manitoba Tomorrow Project cohort. Additionally, we employed other recruitment methods, including sending newsletters to participants who had previously completed the survey and consented to being recontacted. These newsletters encouraged participants to invite their families and friends to take the survey via our online platforms.

### 2.5. Analysis 

Exclusion criteria included age younger than 16 years and incomplete survey responses. Thus, 3352 individuals from Manitoba were recruited into the study, with 5 participants excluded due to their age. Participants could not submit the survey until all questions had been answered on the online platform. Overall, 3347 participants were included in the final analysis. While the survey included a total of 42 questionnaire items, this analysis only utilized data from 23 of the questions, including demographics and questions on UV exposure and skin cancer history, sun protection, and level of worry. 

Survey responses of categorical variables were reported as frequencies or percentages, while continuous variables were reported as means and their accompanying standard deviation. Subgroup analyses were performed by dividing participants based on demographic factors, which were dichotomized, as previously reported [12], e.g., by gender (women vs. men), Fitzpatrick skin phototype (I–III vs. IV–VI), education (university degree or above vs. no university degree), annual household income after tax (≥CAD 50,000 vs. <CAD 50,000), and age (18–49 years vs. ≥50 years). Skin of color was defined as Fitzpatrick skin phototypes IV to VI, as previously reported [28]. All of the studied outcome variables were categorical, and variables that had multiple answer choices were dichotomized as shown in Table 1 and the Supplementary Tables. For example, sun protection methods (e.g., sunscreen use) were dichotomized as always/often vs. never/rarely/sometimes. Logistic regression models were used to calculate age- and gender-adjusted odds ratios and their corresponding 95% confidence intervals and *p*-values. Finally, *p*-values < 0.05 were considered statistically significant. 

## 3. Results

### 3.1. Participants and Descriptive Data

As of March 2024, 3347 Manitoba-based/resident participants completed the survey, with a mean age of 55.7 years and age range of 18 to 84 years. Females constituted the majority of respondents (70.8%), while 4.2% of participants identified as LGBTQ2S+. The majority of participants identified as non-Hispanic White or Euro-Canadian (90.6%). Most participants were of Fitzpatrick skin types II and III (30.2% and 42.3%, respectively). Almost half of the respondents reported an annual income upwards of CAD 90,000 (45.8%), while the highest level of education attained was highly variable. Complete details on this cohort are available in Table 1. 

### 3.2. Overall Sun Habits, Skin Cancer Risk Factors, and Level of Worry

Of the 3347 participants who completed the survey, 285 (8.5%) had a personal history of skin cancer, namely, melanoma (23.9%), basal cell carcinoma (53.7%), and squamous cell carcinoma (12.6%), while 28.2% of participants had a family history of skin cancer. The majority of participants reported a history of lifetime sunburns and lifetime blistering sunburns (67.7% and 67.2%, respectively), while 52.0% reported having used a tanning bed at least once. High or very high total sun exposure was reported by 20.4% of participants, and relatively more participants reported high or very high recreational sun exposure (23.6%), while only 3.8% reported extensive occupational sun exposure. Despite scientific recognition of the dangers associated with UV radiation, tanning and other high-risk sun exposure behaviors continue to predominate. Tanning in the last 12 months was reported by 78.9% of participants, while 31.8% of participants reported spending time in the sun regularly to get a tan or to feel good while on vacation, and 13.5% sunbathed during non-vacation periods as well. Regular use of sun protection was most commonly achieved using sunglasses (70.5% of participants), while the least common sun protection methods were hat wearing (31.0%) and shade seeking (33.8%). Participants who used sunscreen chose SPF30+ in 81.1% of cases, while broad-spectrum sunscreen was used by 62.3% of participants. However, 29.0% did not know the characteristics of the sunscreen they were using. The majority of individuals (82.9%) performed self-skin checks, and when confronted with a new mole, would have it checked by a friend or family member (44.0%). A minority would visit their family physician (38.2%) to assess a mole. Almost all participants reported that they would be worried if the mole was irregular in shape (95.8%), changed color (97.0%), or grew in size (97.7%). Table 1 provides full details for sun habits, skin cancer risk factors, and level of worry across the population sampled. 

### 3.3. Participants’ Knowledge and Reactions

Only 60% of participants disagreed or strongly disagreed that having a base tan is protective against UV radiation and skin damage, while approximately half of the participants disagreed or strongly disagreed that it is rare to get melanoma before the age of 35 (54.4%). When asked whether the participants check their skin for moles on a regular basis, only 66.1% agreed or strongly agreed, highlighting a significant lack of sun awareness and skin cancer risk perception in the studied population. The proportion of individuals agreeing and disagreeing as to whether sunscreens pollute the oceans was similar (26.9% and 27.3%, respectively). More participants disagreed or strongly disagreed that sunscreens contain toxic ingredients (38.0%) compared to those who agreed or strongly agreed (20.5%). Unfortunately, a majority of individuals continue to believe that they look better and/or healthier with a tan (52.3%), while the majority disagree that tanning beds are a safer alternative to natural UV radiation from the sun (77.1%). Full details are provided in Table S1.

### 3.4. Women vs. Men Participants 

**Figure 1:**
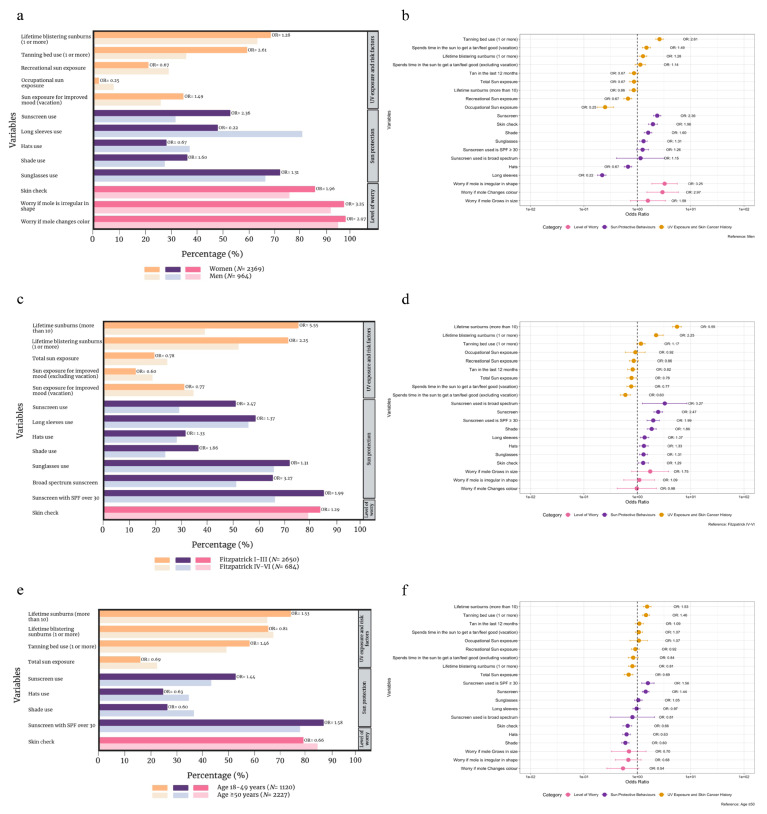
Comparison of sun exposure, melanoma risk factors, sun protection habits, and level of worry for melanoma between women (*n* = 2369) and men (*n* = 964), depicted as a bar graph (**a**) and corresponding forest plot (**b**), between Fitzpatrick skin phototypes I-III (*n* = 2650) and phototypes IV-VI (*n* = 684) as a bar graph (**c**) and corresponding forest plot (**d**), and between ages 18 and 49 years (*n* = 1120) vs. individuals aged ≥50 years (*n* = 2227) as a bar graph (**e**) and corresponding forest plot (**f**). Odds ratios (OR) are adjusted for age and gender where appropriate.

Sun exposure habits, skin cancer risk factors, and level of worry were compared amongst women (*n* = 2369) and men (*n* = 964). Women reported significantly more blistering sunburns over the course of their lives (OR 1.28, *p* = 0.004), greater tanning bed usage (OR 2.61, *p* < 0.001), and more time spent in the sun on a frequent basis during vacation periods to get a tan or to feel good (OR 1.49, *p* < 0.001). Conversely, women were significantly less likely to have high or very high degrees of recreational (OR 0.67, *p* < 0.001) or occupational (OR 0.25, *p* < 0.001) sun exposure. With regards to sun protection, women were significantly more likely to use sunscreen (OR 2.36, *p* < 0.001), wear sunglasses (OR 1.31, *p* < 0.001), and seek shade (OR 1.60, *p* < 0.001), while they wore less long sleeves (OR 0.22, *p* < 0.001) and hats (OR 0.67, *p* < 0.001). Self-skin checks were also more likely to be performed by women as opposed to men (OR 1.96, *p* < 0.001), and women were significantly more likely to worry if a mole was irregular in shape (OR 3.25, *p* < 0.001) or changed color (OR 2.97, *p* = 0.002). A comprehensive comparison is provided in Table S2 and Figure 1a,b.

### 3.5. Fitzpatrick Skin Phototypes I–III vs. IV–VI

Variables were compared amongst individuals of Fitzpatrick skin phototypes I–III (*n* = 2650, lighter skin) and IV–VI (*n* = 684, darker skin). Individuals with lighter skin phototypes (Fitzpatrick skin phototypes I–III) were significantly more likely to have had more than 10 lifetime sunburns (OR 5.55, *p* < 0.001) and 1 or more blistering sunburns (OR 2.25, *p* < 0.001) compared to respondents with skin of color, defined as Fitzpatrick skin phototypes IV–VI. Remarkably, high or very high total sun exposure was significantly lower in lighter skin phototypes (OR 0.78, *p* = 0.02), as was time spent in the sun to get a tan or to feel good, whether at home (OR 0.60, *p* < 0.001) or on vacation (OR 0.77, *p* = 0.005). Often or consistent sun protection was significantly greater in Fitzpatrick phototypes I-III, with sunscreen (OR 2.47, 0 < 0.001), long sleeves (OR 1.37, *p* < 0.001), hats (OR 1.33, *p* = 0.004), shade seeking (OR 1.86, *p* < 0.001), and sunglasses (OR 1.31, *p* = 0.003). Sunscreen was more frequently noted to be broad spectrum (OR 3.27, *p* = 0.02) and SPF30+ (OR 1.99, *p* < 0.001) in lighter skin phototypes. Finally, Fitzpatrick types I–III individuals were significantly more likely to perform regular skin checks (OR 1.29, *p* = 0.02). Table S3 and Figure 1c,d summarize all the findings.

### 3.6. Participants with vs. without a University Education

**Figure 2:**
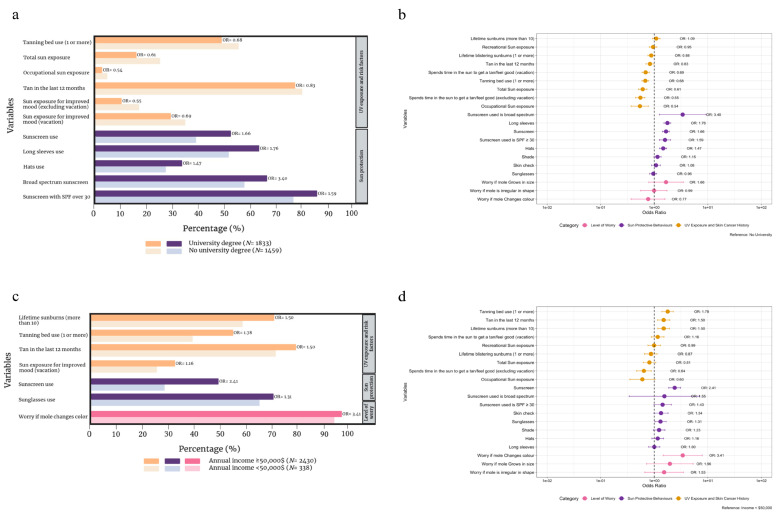
Comparison of sun exposure, melanoma risk factors, sun protection habits, and level of worry for melanoma between those that have completed a university degree (*n* = 1833) and those that have not completed a university degree (*n* = 1459) (**a**) and corresponding forest plot (**b**), and between individuals with an annual income ≥CAD 50,000 (*n* = 2430) and individuals with an annual income <CAD 50,000 (*n* = 338) as a bar graph (**c**) and corresponding forest plot (**d**). Odds ratios (OR) are adjusted for age and gender where appropriate.

Participants with and without a university education were compared. Several damaging sun habits were less frequent amongst those with a university education (*n* = 1833), namely, tanning bed usage (OR 0.68, *p* < 0.001), high or very high total (OR 0.61, *p* < 0.001) or occupational (OR 0.54, *p* = 0.001) sun exposure, tan in the last 12 months (OR 0.83, *p* = 0.04), and time spent outside to get a tan or to feel good during vacation (OR 0.69, *p* < 0.001) or non-vacation periods (OR 0.55, *p* < 0.001). Sun protection was also significantly higher in those with university education with regards to usage of sunscreen (OR 1.66, *p* < 0.001), long sleeves (OR 1.76, *p* < 0.001), and hats (OR 1.47, *p* < 0.001). Both broad spectrum (OR 3.40, *p* = 0.02) and SPF30+ (OR 1.59, *p* < 0.001) usage was greater amongst university-educated individuals. No significant differences were observed with regards to the level of worry between the two groups. Detailed data are provided in Table S4 and Figure 2a,b.

### 3.7. Annual Income ≥ CAD 50,000 vs. <CAD 50,000

Participants who earned over CAD 50,000 annually (after taxes) reported significantly more lifetime sunburns (OR 1.50, *p* < 0.001), greater tanning bed usage (OR 1.78, *p* < 0.001), and tans in the last 12 months (OR 1.50, *p* = 0.003). On the other hand, they spent less time in the sun during non-vacation periods with the goal of tanning (OR 0.64, *p* = 0.005) than those who earned less than CAD 50,000. Sunscreen (OR 2.41, *p* < 0.001) and sunglasses (OR 1.31, *p* = 0.03) were significantly more likely to often or always be worn in higher earners, while differences in other sun protective modalities did not achieve statistical significance. Finally, higher-income participants were significantly more likely to be worried if a mole changed in color (OR 3.41, *p* = 0.004). Table S5 and Figure 2c,d provide a full summary of all compared variables.

### 3.8. Age-Based Comparison

Variables were compared amongst participants aged 18–49 years (*n* = 1120) and those over the age of 50 years (*n* = 2227). Sun exposure variables were rather split amongst the compared groups, with individuals aged 18–49 years reporting more lifetime sunburns (OR 1.53, *p* < 0.001) and tanning bed usage (OR 1.46, *p* < 0.001). On the other hand, they reported less lifetime blistering sunburns (OR 0.81, *p* = 0.007) and high or very high total sun exposure (OR 0.69, *p* < 0.001) compared to those above the age of 50 years. Younger individuals were statistically more likely to use sunscreen (OR 1.44, *p* < 0.001), while they wore less hats (OR 0.63, *p* < 0.001) and sought less shade (OR 0.60, *p* < 0.001). SPF30+ was more likely to be applied by younger participants (OR 1.58, *p* < 0.001); however, they performed less skin checks (OR 0.66, *p* < 0.001). Importantly, behavior patterns did not vary significantly by age in our cohorts, which is concerning since CM incidence rates continue to increase. See Table S6 and Figure 1e,f for a complete analysis of age-related variables.

## 4. Discussion

Our study provided a comprehensive overview of sun protective habits, sun-seeking behaviors, skin cancer risk factors, and level of worry in Manitoba, an important agricultural region in Canada and a representative province of the Canadian prairies, and we elicited statistically significant differences between subgroups of this population. Most importantly, our study identified concerning behavior patterns with regards to safe sun practices. Over half of the participants continue to believe that they look better and/or healthier with a tan. Multiple studies have found that despite acknowledgement of tanning’s associated dangers (even beyond skin cancer), attitudes pertaining to self-perception are stronger driving forces for tanning, and tanning-related sociocultural experiences are important elements in an individual’s desire to have tanned skin [29,30,31,32]. Such beliefs derive from the media-propagated messages of the 1980s, with individuals having tanned skin being portrayed and perceived as more attractive and healthier than their non-tanned counterparts. Such themes continue to predominate today, with individuals who frequent tanning salons indicating improved physical appearance, social acceptance, increased confidence, and happiness as key drivers behind their desire to be tanned [33]. Thus, public health campaigns should aim at shifting social perceptions that view tanned skin as a symbol of health and beauty and instead reinforce the notion that everyone should embrace their natural skin tone. The media plays an instrumental role in tackling this issue, considering that studies have proven its involvement in the marketing of hazardous behaviors, including promoting tanned skin [34]. Importantly, understanding how the media portrays skin cancer is invaluable in the promotion of prevention and education, providing feedback to both government agencies and cancer prevention organizations and allowing for the creation of targeted campaigns [35].

Based on our analysis, while women reported more blistering sunburns, greater tanning bed usage, and more time spent in the sun during vacations or to feel good, they were more inclined to perform skin checks and were more worried if a mole changed or was irregular in shape. Men were more likely to have recreational and occupational sun exposure and were less likely to use sunscreen, wear sunglasses, or seek shade. However, they more often used long-sleeved clothing and hats. Notably, our research group previously elucidated that both CM incidence and mortality rates are worse among Canadian men than they are in women [1,4]. Thus, the behaviors observed through this study partially explain these populational trends. Efforts should focus on raising awareness in both genders, with targeted messages for women focusing on avoiding sunburns and tanning for pleasure, and those for men educating about the risks of recreational and occupational sun exposure. For both genders, emphasizing the use of a combination of clothing and sunscreen is key in achieving clinically effective sun protection. Based on our data, both genders would also benefit from education on the characteristics of skin cancer (e.g., changing or irregular new moles).

With regards to the impact of annual income on sun protective behaviors, the wealthier population was more likely to use sun protection, likely helped by their financial liberty to purchase such products, but they were also more likely to tan on vacation with the goal of tanning for pleasure, to develop a suntan, and to use tanning salons. Individuals who earned less than CAD 50,000 annually wore significantly less sunscreen; however, there were no observed differences with regards to the sunscreen being SPF30+ or broad spectrum. Considering the association between lower income and overall decreased sunscreen usage, we hypothesize that poor utilization is secondary to accessibility issues, as opposed to a lack of awareness, especially given that differences between the high- and low-income groups were attenuated for less costly sun protective modalities, such as long sleeves, hats, and shade seeking. A 2023 study conducted by Melanoma Focus, a charity based in the United Kingdom, found that 50% of individuals think that sunscreen is too expensive, 67% report they would use sunscreen more frequently if it was less costly, and 10% do not use sunscreen at all on the basis of its cost [36]. A recent German study also found that the price of sunscreen was a barrier to its use, among factors such as inconvenience, a lack of perceived need, peer influence, and portrayal as an effeminate person [37]. Overall, advocacy at the level of the provincial and federal governments needs to occur with regards to the control of sunscreen prices and reduction of associated taxes considering their necessity in avoiding ultimately preventable cancers [38,39]. Our recent work highlights that Canada is lagging behind several countries, including Australia, New Zealand, and the United States, whereby it does not provide sales tax exemptions for sunscreen (even when prescribed by a physician) and does not offer tax credits to employers to purchase necessary sun protective equipment [11].

University education also proved to be a statistically significant protective factor with regards to UV exposure and sun protective practices, with participants reporting decreased tanning bed usage, high or very high sun exposure, tans in the last year, and time spent outside with the goal of getting a tan or feeling good, and increased use of sunscreen, long sleeves, hats, and broad-spectrum or SPF30+ sunscreen. Such findings are rather consistent with our group’s previous survey study of Atlantic Canada, which concluded that highly educated individuals were statistically more likely to implement sun protective measures and less likely to use tanning beds [12]. However, as mentioned above, these individuals had high or very high sun exposure and pursued tanning for pleasure. Additionally, studies conducted in other countries have also found that higher levels of education were protective against poor sun protective practices [40], and that those with a college or university degree were more likely to wear sunscreen with a high SPF than participants with lower education levels [41]. Despite these protective factors, higher educational levels have also been associated with increased annual sun exposure and lifetime sunburns in certain populations [12,41]. These findings suggest the existence of a sunscreen paradox, whereby there is a relationship between increased sunscreen use and higher rates of sun exposure, conferring a false sense of security amongst many individuals who solely rely on this method of sun protection [42,43]. Sunscreens are frequently applied in insufficient amounts (usually between 20% and 50% of the recommended 2 mg/cm^2^) and unevenly across the skin. Additionally, a median of 20% of the body surface area is missed during a single application [44,45]. While evidence shows that sunscreen can maintain its protective effect for 8 h after application (as long as swimming, sweating, friction, or other activities that may remove sunscreen are avoided), reapplying sunscreen can compensate for underapplication and missed areas [45,46]. Based on the combination of these findings, more education is needed on the proper application of sunscreen. In addition, a shift in the population’s understanding of sun protection will be crucial to reduce skin cancer rates in the future, whereby we must reorient our messaging from encouraging the use of sunscreen as a sole method of sun protection to a more comprehensive approach, combining sunscreen, sun protective clothing, shade seeking, and sun avoidance during peak sun exposure hours. Importantly, campaigns should reinforce the notion that the only reliable measure of sun protection effectiveness is not getting a tan or sunburn while outdoors and strengthen the idea that no tan is a healthy tan. In addition, public health initiatives need to stress the importance of proper sunscreen application and reapplication, as well as the notion that sunscreen alone does not confer adequate protection from UV radiation.

Individuals with Fitzpatrick phototypes IV-VI were noted to have higher total lifetime sun exposure and were likely to spend more time in the sun with the goal of getting a tan or to feel good. Importantly, public health campaigns should target this subgroup of the population, considering that these high-risk behaviors are likely contributing to the increasing skin cancer morbidity and mortality of this group, in addition to increased photoaging, appearance of melasma, and other sun-associated conditions. Additionally, participants with skin of color used significantly less sun protective modalities and performed fewer skin checks. These findings are in concordance with studies conducted in other countries that reported that individuals with skin of color are significantly less likely to use sunscreen or other forms of sun protection [47,48,49,50,51]. False beliefs continue to persist regarding skin cancer in skin of color, whereby individuals believe that darker skin confers protection against UV rays and that individuals with a dark phototype cannot get skin cancer [52,53]. Such important misconceptions discourage individuals with skin of color from engaging in adequate sun protective practices [54]. Overall, public health initiatives and sun protection campaigns should target these important fallacies to encourage proper sun-safe behaviors in this population.

Finally, the number of individuals who were unaware of the type of sunscreen they wore was remarkable. While 2084 of 3347 participants responded that they used broad-spectrum sunscreen, 29% (*n* = 971) answered that they did not know whether their sunscreen had a broad-spectrum coverage (UVA and UVB) or not. This further contributes to the sunscreen paradox, with individuals relying on the product to prevent skin cancer without knowing whether the sunscreen used is appropriate to achieve this objective. Moreover, sunscreen labels continue to be frequently misunderstood, and often leave shoppers confused or overwhelmed, resulting in misconceptions and an overreliance on the SPF value alone [55,56]. Thus, advocacy for better labeling of sunscreen to promote the visibility of broad-spectrum coverage, in addition to sun safety campaigns reinforcing the necessity of both UVA and UVB protection, would likely result in better comprehension and usage of broad-spectrum sunscreens in the future.

### Strengths and Limitations

With regards to strengths, this survey study provided a detailed analysis of sun protective habits, sun-seeking behaviors, skin cancer risk factors, and level of worry in Manitoba. The in-depth analysis included a large cohort of participants and assessed a wide variety of variables through a modified, validated patient questionnaire, and was able to comment on the statistical significance of findings. Notably, our study was able to comment on findings across the skin phototype spectrum, addressing important concerns with regards to sun protective practices in individuals with skin of color. Concerning the study’s limitations, due to the nature of survey studies, our results were subject to both recall and participation biases. In addition, the survey did not assess certain risk factors, such as marital status. Notably, a study found a 35% reduction in CM mortality risk among married individuals, which was attributed not only to earlier detection but also to factors such as social support [57].

## 5. Conclusions

The incidence of skin cancer in Canada is increasing in a linear fashion, where soon one-third of Canadians will be affected by a skin cancer in their lifetime. Within this context, our Manitoba-based study highlighted a concerning persistence of high-risk sun exposure behaviors in combination with a lack of skin cancer awareness amongst Canadians. Specifically, over 65% of individuals reported a history of sunburns, including blistering sunburns, more than 50% had used a tanning bed, over 75% had tanned for pleasure in the last 12 months, and nearly one-third of the population reported having spent time in the sun regularly for pleasure as well as feeling good while tanning. Over 40% of individuals agreed or felt neutral that having a base tan is protective, while nearly 50% felt that CM is rare in individuals under 35 years of age. Most troubling is that over 50% of individuals continue to believe that they look better and/or healthier with a tan. The perpetual existence of such behaviors and attitudes may result in ineffective sun protective measures and, in turn, the acceleration of skin cancer incidence rates unless the population begins to recognize to what degree tanning and sunburns are high-risk, dangerous activities with serious health consequences. Furthermore, the combination of such harmful behaviors and attitudes with inadequate sun protection efforts (e.g., sun protective clothing use less than 60% of the time and sunscreen use less than 50% of the time) only further perpetuates the issue at hand, and reinforces the need to implement targeted, effective public health campaigns. Moreover, in those who applied sunscreen, ~30% were unaware of its characteristics and whether it was suitable to prevent sunburns, skin cancer, or both. Regarding early cancer detection, concerningly, only ~1/3 of the population reported that they would consult a family physician to assess a changing mole.

In addition to these general overall worrisome trends, our study highlighted that sun exposure behaviors and skin cancer concern varied according to different subgroups of the Manitoba/Canadian population. Notably, skin cancer awareness was generally lower and sun exposure patterns were higher in darker skin individuals. Men were reluctant to use sunscreen, and wealthier and university-educated individuals were more likely to engage in recreational sun exposure. Occupational exposure was more common in less affluent individuals, who were also less likely to be able to afford sunscreen and cost-associated sun protection measures. For these reasons, the development of targeted public health campaigns to modify current behavior trends and attitudes among the Canadian public, coupled with government/community interventions making sun protection more affordable/available, is quintessential in the fight to decrease skin cancer morbidity and mortality in years to come.

Specifically, we recommend curated social media, television, and print advertisements established by the Canadian government and cancer prevention organizations reinforcing the importance of a comprehensive sun protection approach, including sun protective clothing, sunscreen, shade seeking, and sun avoidance during peak hours for all, as well as gender-specific recommendations based on observed differences, such as reinforcing sunscreen usage, skin checks, and shade seeking for men, all while embracing the idea that sun protection is not emasculating. Moreover, advocacy needs to occur at all levels of government to make sunscreens more affordable for all, whether through price control and/or elimination of sales taxes. Finally, we hope that proper sun safety techniques will someday be taught in school, so that healthy lifelong habits can be established early on to limit the incidence of skin cancers in years to come.

## Figures and Tables

**Table 1:** Population demographic characteristics, ultraviolet exposure, melanoma risk factors, sun protection habits, and level of worry for melanoma. Individuals that answered ‘I do not know’ or ‘I would rather not say’ were not included in the table. In total, 3347 participants completed the survey.

Variable	*n* (%)
Mean age (SD)	55.7 (12.4)
Median age (range)	58 (18–84)
Gender	
Men	964 (28.8)
Women	2369 (70.8)
Other	14 (0.4)
LGBTQ2S+	139 (4.2)
Ethnicity	
Non-Hispanic White or Euro-Canadian	3034 (90.6)
Other	313 (9.4)
Annual income (CAD)	
<20,000	35 (1.0)
20,000–49,999	303 (9.1)
50,000–69,999	409 (12.2)
70,000–89,999	489 (14.6)
≥90,000	1532 (45.8)
Highest level of education completed	
No high school	31 (0.9)
High school	538 (16.1)
Junior college or associate degree	890 (26.6)
University bachelor’s degree	1181 (35.3)
Graduate or doctoral studies	652 (19.5)
Fitzpatrick	
Type I	225 (6.7)
Type II	1010 (30.2)
Type III	1415 (42.3)
Type IV	573 (17.1)
Type V	106 (3.2)
Type VI	5 (0.1)
Personal history of skin cancer	285 (8.5)
Melanoma	68 (23.9)
Squamous cell carcinoma (SCC)	36 (12.6)
Basal cell carcinoma (BCC)	153 (53.7)
Family history of skin cancer	943 (28.2)
Lifetime sunburns (more than 10)	2267 (67.7)
Lifetime blistering sunburns (1 or more)	2250 (67.2)
Tanning bed use (1 or more)	1741 (52.0)
Sun exposure (‘high’ or ‘very high’)	
Total	682 (20.4)
Recreational	788 (23.6)
Occupational	128 (3.8)
Tan in the last 12 months	2641 (78.9)
Spends time in the sun daily or multiple days per week to get a tan or to feel good (excluding vacation)	453 (13.5)
Spends time in the sun daily or multiple days per week to get a tan or to feel good (vacation)	1061 (31.8)
Sun protection (‘often’ or ‘always’)	
Sunscreen	1555 (46.5)
Long sleeves	1932 (57.7)
Hats	1038 (31.0)
Shade	1131 (33.8)
Sunglasses	2361 (70.5)
Sunscreen	
Broad spectrum	2084 (62.3)
SPF ≥ 30	2714 (81.1)
Skin check	2775 (82.9)
Reaction to a new mole	
Family doctor visit	1278 (38.2)
Check by friend/family member	1472 (44.0)
Ignore	186 (5.6)
Search the internet	305 (9.1)
Worry if mole	
Is irregular in shape	3206 (95.8)
Changes color	3246 (97.0)
Grows in size	3270 (97.7)

## Data Availability

All available compiled data are presented in the text of the manuscript and in the Supplementary Materials. Deidentified participant data, data dictionaries, and the study protocol are available upon request with publication.

## Supplementary Materials

Supplemental Tables S1–S6. The following supporting information can be downloaded at: https://www.mdpi.com/article/10.3390/cancers16173093/s1. Table S1: Participants’ reactions to various quotes. Individuals that answered ‘I do not know’ or ‘I would rather not say’ were not included in the table. In total, 3347 participants completed the survey. Table S2: Comparison of sun exposure, melanoma risk factors, sun protection habits and level of worry for melanoma between women (*n* = 2369) vs. men (*n* = 964). Individuals that answered ‘I do not know’ or ‘I would rather not say’ were not included in the analysis. The odds ratios (OR) are adjusted for age and gender. Statistically significant ORs are highlighted with a star (*). Table S3: Comparison of sun exposure, melanoma risk factors, sun protection habits and level of worry for melanoma between individuals with a Fitzpatrick skin phototypes I-III (*n* = 2650) vs. phototypes IV-VI (*n* = 684). Individuals that answered ‘I do not know’ or ‘I would rather not say’ were not included in the analysis. The odds ratios (OR) are adjusted for age and gender. Statistically significant ORs are highlighted with a star (*). Table S4: Comparison of sun exposure, melanoma risk factors, sun protection habits and level of worry for melanoma between those that have completed a university degree (*n* = 1833) vs. those that have not completed a university degree (*n* = 1459). Individuals that answered ‘I do not know’ or ‘I would rather not say’ were not included in the analysis. The odds ratios (OR) are adjusted for age and gender. Statistically significant ORs are highlighted with a star (*). Table S5: Comparison of sun exposure, melanoma risk factors, sun protection habits and level of worry for melanoma between individuals with an annual income ≥50,000$ (*n* = 2430) vs. individuals with an annual income <50,000$ (*n* = 338). Individuals that answered ‘I do not know’ or ‘I would rather not say’ were not included in the analysis. The odds ratios (OR) are adjusted for age and gender. Statistically significant ORs are highlighted with a star (*). Table S6: Comparison of sun exposure, melanoma risk factors, sun protection habits and level of worry for melanoma between individuals between the ages 18–49 years (*n* = 1120) vs. individuals age ≥50 years (*n* = 2227). Individuals that answered ‘I do not know’ or ‘I would rather not say’ were not included in the analysis. The odds ratios (OR) are adjusted for age and gender. Statistically significant ORs are highlighted with a star (*).
