# Supplementary material for: A Comprehensive Analysis of Skin Cancer Concerns and Protective Practices in Manitoba, Canada, Highlights Lack of Skin Cancer Awareness and Predominance of High-Risk Sun Exposure Behaviors
Source: Cancers (Basel). 2024 Sep 5;16(17):3093. doi: 10.3390/cancers16173093 (PMC11394652; doi:10.3390/cancers16173093)
Supplement: Supplementary file 1 [file cancers-16-03093-s001.zip › cancers-3148723-supplementary.pdf]

Table S1. Participants' reactions to various quotes. Individuals that answered 'I do not know' or 'I would rather not say' were not included in the table. In total, 3347 participants completed the survey.

Quote	N (%)
"Having a base tan is protective against the sun's UV radiation/skin damage"	
Agree and strongly agree	505 (15.1)
Disagree and strongly disagree	2009 (60.0)
"It is rare to get melanoma before the age of 35"	
Agree and strongly agree	436 (13.0)
Disagree and strongly disagree	1820 (54.4)
"I check my skin on a regular basis for abnormal moles"	
Agree and strongly agree	2214 (66.1)
Disagree and strongly disagree	583 (17.4)
"Sunscreens pollute the oceans"	
Agree and strongly agree	901 (26.9)
Disagree and strongly disagree	915 (27.3)
"Sunscreens contain toxic ingredients"	
Agree and strongly agree	687 (20.5)
Disagree and strongly disagree	1273 (38.0)
"I look better and/or healthier with a tan"	
Agree and strongly agree	1749 (52.3)
Disagree and strongly disagree	705 (21.1)
"Tanning beds are a safer, more controlled way to get a tan than from the sun"	
Agree and strongly agree	110 (3.3)
Disagree and strongly disagree	2582 (77.1)

Table S2. Comparison of sun exposure, melanoma risk factors, sun protection habits and level of worry for melanoma between women (n=2369) *vs.* men (n=964). Individuals that answered 'I do not know' or 'I would rather not say' were not included in the analysis. The odds ratios (OR) are adjusted for age and gender. Statistically significant ORs are highlighted with a star (*).

**Variable**	**Women N (%)**	**Men N (%)**	**Adjusted OR (95% CI)**	**p-value**
Lifetime sunburns (more than 10)	1586 (66.9)	672 (69.7)	0.86 (0.72-1.02)	0.08
Lifetime blistering sunburns (1 or more)	1629 (68.8)	608 (63.1)	1.28 (1.08-1.51)	0.004*
Tanning bed use (1 or more)	1400 (59.1)	341 (35.4)	2.61 (2.23-3.05)	<0.001*
Sun exposure ('high' or 'very high')				
Total	467 (19.7)	215 (22.3)	0.87 (0.72-1.04)	0.14
Recreational	508 (21.4)	279 (28.9)	0.67 (0.56-0.79)	<0.001*
Occupational	51 (2.2)	77 (8.0)	0.25 (0.18-0.36)	<0.001*
Tan in the last 12 months	1858 (78.4)	774 (80.3)	0.87 (0.72-1.06)	0.16
Spends time in the sun daily or multiple days per week to get a tan or to feel good (excluding vacation)	332 (14.0)	121 (12.6)	1.14 (0.91-1.42)	0.26
Spends time in the sun daily or multiple days per week to get a tan or to feel good (vacation)	813 (34.4)	247 (25.8)	1.49 (1.25-1.77)	<0.001*
Sun protection ('often' or 'always')				
Sunscreen	1242 (52.4)	303 (31.4)	2.36 (2.01-2.77)	<0.001*
Long sleeves	1139 (48.1)	783 (81.2)	0.22 (0.18-0.26)	<0.001*
Hats	670 (28.3)	363 (37.7)	0.67 (0.57-0.79)	<0.001*
Shade	860 (36.3)	262 (27.2)	1.60 (1.35-1.88)	<0.001*
Sunglasses	1711 (72.2)	641 (66.5)	1.31 (1.11-1.54)	0.001*
Sunscreen				
Broad spectrum	1543 (65.1)	530 (55.0)	1.15 (0.41-3.23)	0.80
SPF $\geq$ 30	1998 (84.3)	704 (73.0)	1.26 (0.97-1.62)	0.08
Skin check	2032 (85.8)	732 (75.9)	1.96 (1.62-2.38)	<0.001*
Worry if mole				
Is irregular in shape	2306 (97.4)	886 (91.9)	3.25 (1.88-5.62)	<0.001*
Changes colour	2321 (98.0)	911 (94.5)	2.97 (1.51-5.87)	0.002*
Grows in size	2330 (98.4)	926 (96.1)	1.58 (0.74-3.36)	0.24

Table S3. Comparison of sun exposure, melanoma risk factors, sun protection habits and level of worry for melanoma between individuals with a Fitzpatrick skin phototypes I-III (n=2650) *vs.* phototypes IV-VI (n=684). Individuals that answered 'I do not know' or 'I would rather not say' were not included in the analysis. The odds ratios (OR) are adjusted for age and gender. Statistically significant ORs are highlighted with a star (*).

Variable	Fitzpatrick I-III N (%)	Fitzpatrick IV-VI N (%)	Adjusted OR (95% CI)	p-value
Lifetime sunburns (more than 10)	1997 (75.4)	267 (39.0)	5.55 (4.60-6.69)	<0.001*
Lifetime blistering sunburns (1 or more)	1888 (71.2)	357 (52.2)	2.25 (2.13-3.05)	<0.001*
Tanning bed use (1 or more)	1419 (53.5)	318 (46.5)	1.17 (0.98-1.40)	0.08
Sun exposure ('high' or 'very high')				
Total	516 (19.5)	165 (24.1)	0.78 (0.64-0.96)	0.02*
Recreational	606 (22.9)	180 (26.3)	0.86 (0.71-1.05)	0.13
Occupational	96 (3.6)	31 (4.5)	0.92 (0.60-1.40)	0.70
Tan in the last 12 months	2078 (78.4)	556 (81.3)	0.82 (0.66-1.02)	0.08
Spends time in the sun daily or multiple days per week to get a tan or to feel good (excluding vacation)	326 (12.3)	127 (18.6)	0.60 (0.48-0.75)	<0.001*
Spends time in the sun daily or multiple days per week to get a tan or to feel good (vacation)	821 (31.1)	240 (35.2)	0.77 (0.64-0.92)	0.005*
Sun protection ('often' or 'always')				
Sunscreen	1354 (51.1)	193 (28.2)	2.47 (2.05-2.98)	<0.001*
Long sleeves	1544 (58.3)	378 (55.3)	1.37 (1.14-1.65)	<0.001*
Hats	843 (31.8)	191 (27.9)	1.33 (1.10-1.60)	0.004*
Shade	962 (36.3)	163 (23.8)	1.86 (1.53-2.27)	<0.001*
Sunglasses	1902 (71.8)	447 (65.4)	1.31 (1.10-1.57)	0.003*
Sunscreen				
Broad spectrum	1724 (65.1)	351 (51.3)	3.27 (1.25-8.52)	0.02*
SPF ≥ 30	2253 (85.0)	450 (65.8)	1.99 (1.52-2.60)	<0.001*
Skin check	2223 (83.9)	544 (79.5)	1.29 (1.04-1.61)	0.02*
Worry if mole				
Is irregular in shape	2555 (96.5)	640 (93.6)	1.09 (0.56-2.10)	0.80
Changes colour	2580 (97.4)	654 (95.6)	0.98 (0.42-2.28)	0.96
Grows in size	2596 (98.0)	662 (96.8)	1.75 (0.79-3.89)	0.17

Table S4. Comparison of sun exposure, melanoma risk factors, sun protection habits and level of worry for melanoma between those that have completed a university degree (n=1833) *vs.* those that have not completed a university degree (n=1459). Individuals that answered 'I do not know' or 'I would rather not say' were not included in the analysis. The odds ratios (OR) are adjusted for age and gender. Statistically significant ORs are highlighted with a star (*).

**Variable**	**University N (%)**	**No university N (%)**	**Adjusted OR (95% CI)**	**p-value**
Lifetime sunburns (more than 10)	1272 (69.4)	964 (66.1)	1.09 (0.93-1.28)	0.28
Lifetime blistering sunburns (1 or more)	1212 (66.1)	1003 (68.7)	0.88 (0.76-1.03)	0.12
Tanning bed use (1 or more)	898 (49.0)	814 (55.8)	0.68 (0.59-0.79)	<0.001*
Sun exposure ('high' or 'very high')				
Total	303 (16.5)	366 (25.1)	0.61 (0.51-0.73)	<0.001*
Recreational	423 (23.1)	351 (24.1)	0.95 (0.81-1.12)	0.53
Occupational	51 (2.8)	73 (5.0)	0.54 (0.37-0.78)	0.001*
Tan in the last 12 months	1425 (77.7)	1175 (80.5)	0.83 (0.70-0.99)	0.04*
Spends time in the sun daily or multiple days per week to get a tan or to feel good (excluding vacation)	191 (10.4)	253 (17.3)	0.55 (0.45-0.67)	<0.001*
Spends time in the sun daily or multiple days per week to get a tan or to feel good (vacation)	537 (29.3)	505 (34.8)	0.69 (0.59-0.81)	<0.001*
Sun protection ('often' or 'always')				
Sunscreen	961 (52.4)	570 (39.1)	1.66 (1.43-1.91)	<0.001*
Long sleeves	1155 (63.0)	753 (51.6)	1.76 (1.52-2.04)	<0.001*
Hats	619 (33.8)	403 (27.6)	1.47 (1.26-1.71)	<0.001*
Shade	632 (34.5)	481 (33.0)	1.15 (0.99-1.34)	0.06
Sunglasses	1287 (70.2)	1037 (71.1)	0.96 (0.82-1.11)	0.57
Sunscreen				
Broad spectrum	1222 (66.7)	842 (57.7)	3.40 (1.25-9.29)	0.02*
SPF $\geq$ 30	1567 (85.5)	1114 (76.4)	1.59 (1.25-2.03)	<0.001*
Skin check	1527 (83.3)	1207 (82.7)	1.08 (0.89-1.30)	0.45
Worry if mole				
Is irregular in shape	1764 (96.3)	1392 (95.4)	0.99 (0.56-1.73)	0.96
Changes colour	1784 (97.3)	1412 (96.8)	0.77 (0.37-1.58)	0.47
Grows in size	1797 (98.0)	1420 (97.3)	1.66 (0.79-3.48)	0.18

Table S5. Comparison of sun exposure, melanoma risk factors, sun protection habits and level of worry for melanoma between individuals with an annual income ≥50 000$ (n=2430) *vs.* individuals with an annual income <50,000$ (n=338). Individuals that answered 'I do not know' or 'I would rather not say' were not included in the analysis. The odds ratios (OR) are adjusted for age and gender. Statistically significant ORs are highlighted with a star (*).

**Variable**	**Income ≥50,000$ N (%)**	**Income <50,000$ N (%)**	**Adjusted OR (95% CI)**	**p-value**
Lifetime sunburns (more than 10)	1713 (70.5)	198 (58.6)	1.50 (1.17-1.92)	0.001*
Lifetime blistering sunburns (1 or more)	1640 (67.5)	239 (70.7)	0.87 (0.66-1.13)	0.29
Tanning bed use (1 or more)	1323 (54.4)	133 (39.3)	1.78 (1.39-2.26)	<0.001*
Sun exposure ('high' or 'very high')				
Total	481 (19.8)	82 (24.3)	0.81 (0.62-1.07)	0.14
Recreational	576 (23.7)	79 (23.4)	0.99 (0.76-1.31)	0.96
Occupational	88 (3.6)	18 (5.3)	0.60 (0.35-1.03)	0.07
Tan in the last 12 months	1935 (79.6)	242 (71.6)	1.50 (1.15-1.95)	0.003*
Spends time in the sun daily or multiple days per week to get a tan or to feel good (excluding vacation)	309 (12.7)	61 (18.0)	0.64 (0.47-0.88)	0.005*
Spends time in the sun daily or multiple days per week to get a tan or to feel good (vacation)	786 (32.4)	86 (25.9)	1.16 (0.88-1.52)	0.29
Sun protection ('often' or 'always')				
Sunscreen	1201 (49.4)	97 (28.7)	2.41 (1.86-3.11)	<0.001*
Long sleeves	1422 (58.5)	197 (58.3)	1.00 (0.78-1.27)	0.98
Hats	749 (30.8)	103 (30.5)	1.16 (0.90-1.49)	0.26
Shade	813 (33.5)	110 (32.5)	1.23 (0.96-1.58)	0.10
Sunglasses	1713 (70.5)	219 (64.8)	1.31 (1.03-1.67)	0.03*
Sunscreen				
Broad spectrum	1555 (64.0)	169 (50.0)	1.55 (0.34-6.98)	0.571
SPF ≥ 30	2027 (83.4)	237 (70.1)	1.43 (0.99-2.09)	0.06
Skin check	2012 (82.8)	272 (80.5)	1.34 (0.99-1.81)	0.06
Worry if mole				
Is irregular in shape	2330 (95.9)	321 (95.0)	1.53 (0.67-3.51)	0.31
Changes colour	2365 (97.3)	318 (94.1)	3.41 (1.47-7.89)	0.004*
Grows in size	2376 (97.8)	327 (96.7)	1.96 (0.72-5.33)	0.19

Table S6. Comparison of sun exposure, melanoma risk factors, sun protection habits and level of worry for melanoma between individuals between the ages 18-49 years (n=1120) *vs.* individuals age ≥50 years (n=2227). Individuals that answered 'I do not know' or 'I would rather not say' were not included in the analysis. The odds ratios (OR) are adjusted for age and gender. Statistically significant ORs are highlighted with a star (*).

**Variable**	**Age 18-49 N (%)**	**Age ≥50 N (%)**	**Adjusted OR (95% CI)**	**p-value**
Lifetime sunburns (more than 10)	829 (74.0)	1438 (64.6)	1.53 (1.29-1.81)	<0.001*
Lifetime blistering sunburns (1 or more)	731 (65.3)	1519 (68.2)	0.81 (0.69-0.94)	0.007*
Tanning bed use (1 or more)	650 (58.0)	1091 (49.0)	1.46 (1.26-1.70)	<0.001*
Sun exposure ('high' or 'very high')				
Total	183 (16.4)	499 (22.4)	0.69 (0.57-0.83)	<0.001*
Recreational	250 (22.3)	538 (24.2)	0.92 (0.78-1.09)	0.35
Occupational	43 (3.8)	85 (3.8)	1.07 (0.73-1.56)	0.74
Tan in the last 12 months	893 (79.7)	1748 (78.5)	1.09 (0.91-1.31)	0.36
Spends time in the sun daily or multiple days per week to get a tan or to feel good (excluding vacation)	136 (12.1)	317 (14.2)	0.84 (0.68-1.04)	0.11
Spends time in the sun daily or multiple days per week to get a tan or to feel good (vacation)	363 (32.5)	698 (31.5)	1.07 (0.92-1.26)	0.38
Sun protection ('often' or 'always')				
Sunscreen	591 (52.8)	964 (43.3)	1.44 (1.24-1.66)	<0.001*
Long sleeves	635 (56.7)	1297 (58.2)	0.97 (0.83-1.13)	0.67
Hats	275 (24.6)	763 (34.3)	0.63 (0.53-0.74)	<0.001*
Shade	301 (26.9)	830 (37.3)	0.60 (0.51-0.70)	<0.001*
Sunglasses	798 (71.3)	1563 (70.2)	1.05 (0.89-1.23)	0.56
Sunscreen				
Broad spectrum	714 (63.8)	1370 (61.5)	0.81 (0.31-2.10)	0.66
SPF ≥ 30	976 (87.1)	1738 (78.0)	1.58 (1.21-2.06)	<0.001*
Skin check	889 (79.4)	1886 (84.7)	0.66 (0.54-0.79)	<0.001*
Worry if mole				
Is irregular in shape	1074 (95.9)	2132 (95.8)	0.68 (0.39-1.18)	0.17
Changes colour	1087 (97.1)	2159 (96.9)	0.54 (0.27-1.07)	0.08
Grows in size	1094 (97.7)	2176 (97.7)	0.70 (0.33-1.46)	0.34
